# Inhibition of BACE1 Activity by a DNA Aptamer in an Alzheimer’s Disease Cell Model

**DOI:** 10.1371/journal.pone.0140733

**Published:** 2015-10-16

**Authors:** Huiyu Liang, Yusheng Shi, Zhewen Kou, Yonghua Peng, Wenjun Chen, Xiaowen Li, Shuji Li, Ying Wang, Fang Wang, Xingmei Zhang

**Affiliations:** 1 Key Laboratory of Psychiatric Disorders of Guangdong Province, Department of Neurobiology, School of Basic Medical Sciences, Southern Medical University, Guangzhou, China; 2 Department of Radiation Oncology, Nanfang Hospital, Southern Medical University, Guangzhou, China; 3 Cancer Research Institute, School of Basic Medical Sciences, Southern Medical University, Guangzhou, China; Consiglio Nazionale delle Ricerche (CNR), ITALY

## Abstract

An initial step in amyloid-β (Aβ) production includes amyloid precursor protein (APP) cleavage via β-Site amyloid precursor protein cleaving enzyme 1 (BACE1). Increased levels of brain Aβ have been implicated in the pathogenesis of Alzheimer’s disease (AD). Thus, β-secretase represents a primary target for inhibitor drug development in AD. In this study, aptamers were obtained from combinatorial oligonucleotide libraries using a technology referred to as systematic evolution of ligands by exponential enrichment (SELEX). A purified human BACE1 extracellular domain was used as a target to conduct an *in vitro* selection process using SELEX. Two DNA aptamers were capable of binding to BACE1 with high affinity and good specificity, with *Kd* values in the nanomolar range. We subsequently confirmed that one aptamer, A1, exhibited a distinct inhibitory effect on BACE1 activity in an AD cell model. We detected the effects of M17-APPsw cells that stably expressed Swedish mutant APP after aptamer A1 treatment. Aβ40 and Aβ42 concentrations secreted by M17-APPsw cells decreased intracellularly and in culture media. Furthermore, Western blot analysis indicated that sAPPβ expression significantly decreased in the A1 treated versus control groups. These findings support the preliminary feasibility of an aptamer evolved from a SELEX strategy to function as a potential BACE1 inhibitor. To our knowledge, this is the first study to acquire a DNA aptamer that exhibited binding specificity to BACE1 and inhibited its activity.

## Introduction

Alzheimer’s disease (AD) is a chronic degenerative disease of the central nervous system (CNS), which is primarily manifested by cognitive impairment, particularly memory deterioration. The decline in daily living activities of AD patients, as well as behavioral and psychological symptoms, result in substantial emotional and financial burdens on patients, their families, and society. In recent years, the morbidity of AD has increased as a result of an aging population and increased diagnostic rates, and it has become a more serious healthcare problem [1]. The build-up of amyloid-β (Aβ) peptides in the brain has been linked to AD pathogenesis and may represent a key target for AD modification[2, 3]. Aβ formation occurs via sequential proteolytic processing of amyloid precursor protein (APP) and is catalyzed by β- and γ-secretases.

β-site APP-cleaving enzyme 1 (BACE1) is a membrane-bound aspartic protease and the rate-limiting step in Aβ generation, which is responsible for β-secretase cleavage of APP [4]. Evidence indicates that BACE1 protein levels and activity are upregulated in the brains of sporadic AD patients [5]. Furthermore, increased BACE1 levels have been reported in cerebrospinal fluid (CSF) of prodromal AD patients [6]. Moreover, an increased affinity of APP binding to BACE1 has been reported in patients who carry the Swedish mutation in the APP gene (APPsw), which subsequently increased Aβ production [7]. A coding mutation in the APP gene (APPA673T) located at a site proximal to the BACE1 proteolytic site decreased BACE1 cleavage of APP and was protective against AD, which provides additional evidence that the inhibition of BACE1 cleavage of APP may protect against AD [8, 9]. Previous studies have demonstrated that decreased BACE1 activity altered the amyloid burden in mice [10–14]. Thus, BACE1 represents a promising target for mechanistic-based AD treatment. To date, BACE1 inhibitor development has been very challenging, and no safe and effective BACE1 inhibitor has been used in clinical populations [15].

Aptamers are obtained from combinatorial oligonucleotide libraries using a technology referred to as systematic evolution of ligands by exponential enrichment (SELEX). These single-stranded oligonucleotides are capable of specific and high-affinity binding to target molecules due to their tertiary structures. Compared with conventional antibodies, aptamers have a substantial number of attractive features including low molecular weight, quick and reproducible synthesis *in vitro*, easy modification, good stability, low toxicity, low immunogenicity and rapid tissue penetration [15, 16]. Purified proteins are the most common targets for SELEX selection. Aptamers selected directly against proteins can inhibit target protein activity with high affinity and good specificity [17], and can be used to investigate the mechanisms of interaction between proteins and nucleic acids [18]. These advantages have made aptamers excellent alternatives for diagnostic and therapeutic agents in CNS related pathologies, because antibody-based approaches have not demonstrated adequate sensitivity in most cases, whereas they have exhibited toxicity *in vivo* and the inability to cross the blood-brain barrier (BBB) efficiently. Although several small-molecule BACE1 inhibitors have been developed in AD research [9, 10, 19], there is currently no BACE1 inhibitor available on the market. Therefore, the development of a novel type of BACE1 inhibitor is very important.

This current study used a purified human BACE1 extracellular domain as a target to perform the SELEX process, and obtained two highly efficient and specific aptamers to BACE1 (i.e. A1 and A2). The A1 aptamer decreased Aβ_40_ and Aβ_42_ production, as well as sAPPβ expression, in M17-APPsw cell cultures (AD cell model). These novel findings support the initial potential of A1 as a BACE1 inhibitor for the treatment of AD. To our knowledge, this is the first investigation to acquire a DNA aptamer that exhibits binding specificity to BACE1 and inhibits its activity.

## Materials and Methods

### Cell culture

M17 human neuroblastoma cells that stably expressed Swedish mutant APP (M17-APPsw cells) were a gift from Professor Zhu Xiongwei (Department of Pathology, Case Western Reserve University, Cleveland, Ohio, USA). Cells were maintained in Opti-MEM supplemented with 10% fetal bovine serum (FBS), 100 U/ml of penicillin, 100 ug/ml of streptomycin (P/S), and 20 mg/ml of Geneticin in a 5% CO_2_/95% air atmosphere environment at 37°C.

### Random library, primers and control aptamer

The synthetic single stranded DNA (ssDNA) library consists of a random sequence of 30 nt in the middle and two flanked primer hybridization sites [20]:

5′-GCAATGGTACGGTACTTCC-(N30)-CAAAAGTGCACGCTACTTTGCTAA-3′.

Sense strand primer P1: 5′-GCAATGGTACGGTACTTCC-3′.

Antisense strand primer P2: 5′-TTAGCAAAGTAGCGTGCACTTTTG-3′.

Structured 3′ antisense strand primer P3:

5′-GCTAAGCGGGTGGGACTTCCTAGTCCCACCCGCTTAGCAAAGTAGCGTGCACTTTTG-3′.

P1 and P2 were utilized to synthesize double-stranded DNA (dsDNA). P1 and P3 were used to synthesize ssDNA via asymmetric polymerase chain reaction (PCR). All sequences including biotin labeled ssDNA were manufactured by Invitrogen (Carlsbad, CA, USA).

### SELEX procedure

This SELEX strategy was conducted as previously described with minor modifications [21]. Briefly, the purified human BACE1 extracellular domain (Sigma Aldrich, St. Louis, MO, USA) was coated onto 96-well plates at 4°C overnight, and the wells were subsequently blocked with 5% bovine serum albumin (BSA). The DNA library was heated at 95°C for five minutes and snap-cooled for five minutes on ice. Then, it was maintained at room temperature for 30 minutes in binding buffer (0.45 g of glucose, 10 mg of yeast tRNA, 5 mg of salmon sperm DNA, 0.1 g of BSA, and 0.5 ml of 1 M MgCl_2_ in 100 ml of 1 x phosphate-buffered saline [PBS]) prior to incubation with BACE1. After incubation, the wells were washed six times with washing buffer (0.45 g of glucose and 0.5 ml of 1 M MgCl_2_ in 100 ml of 1 x PBS). The plate was placed on a 95°C heat block for five minutes and maintained on ice for five minutes to dissociate DNA from the target. Since the 4^th^ round of selection by SELEX, the generated ssDNA pool was incubated with a blank well for counter selection. The unbound sequences were subsequently incubated with BACE1. BACE1 protein amount, DNA pool volume, and incubation time were gradually decreased to increase selection stringency, whereas washing time was increased during this process (Table 1).

**Table 1:** Selection conditions in the SELEX process.

Cycle	Amount of BACE1(μg)	ssDNA pool (pmol)	Salmon sperm DNA (μg/μl)	Incubation time (minutes)	washing conditions
1	7	2500	0.05	120	6×1 min
2	5	500	0.1	120	6×1 min
3	2.5	250	0.1	60	6×2 min
4^a^	2.5	250	0.1	40	6×2 min
5^a^	1	250	0.1	40	6×3 min
6^a^	1	250	1	30	6×3 min
7^a^	0.5	250	1	30	6×3 min

BACE1: β-Site amyloid precursor protein cleaving enzyme 1

^a^A blank control well was introduced for counter selection since the 4^th^ round.

### Preparation of ssDNA via asymmetric PCR

Using P1 and P3 primers, the production of ssDNA molecules occurred via asymmetric PCR as follows [22]. One hundred μl of PCR mixture, which contained 10 μl of 10 x PCR buffer, 0.2 mM of dNTPs, 1 μM of each primer, 20 nM of template, and 2.5 U of Taq DNA polymerase, was thermally cycled 30 times at 94°C for one minute, 37°C for 80 seconds, and 58°C for one minute, followed by a final extension for five minutes at 58°C. Unequal length PCR products were analyzed via electrophoresis in an 8% polyacrylamide-7M urea gel, and the lower band of interest was purified. The gel was eluted via the addition of elution buffer (0.5 M of NH_4_Ac, 0.2% SDS, and 1 M of EDTA [pH 8.0]). The ssDNA was collected and precipitated by the addition of 10 mM of MgCl_2_, 0.3 M of NaAc, and 2.5-fold ethanol by volume at -20°C overnight. Following centrifugation, the pellet was rinsed with 70% ethanol and dried at room temperature.

### Binding affinity assay

BACE1 (50 μM per well, diluted in 1 x PBS) was coated onto 96-well plates at 4°C overnight, blocked with 5% BSA at 37°C for 60 minutes, washed with PBST (0.01% Tween 20 in 1 x PBS, pH 7.4), and incubated with different concentrations of biotinylated aptamers at 37°C for 60 minutes. Streptavidin-conjugated horseradish peroxidase (50 μl, 1:1000 in PBS; Sigma Aldrich, St. Louis, MO, USA) was added to the wells and incubated for 30 minutes at 37°C. Fifty ul of TMB solution were added for an additional 20 minutes of incubation. The reaction was terminated with 25 μl of 2 M H_2_SO_4_, and the absorbance was measured at 450 nm. Apparent equilibrium dissociation constant (*K*_d_) values were determined for each aptamer using nonlinear regression (SigmaPlot 12.0; Systat Software, San Jose, CA, USA) according to the following equation: *Y = B*_*max*_ *X/(K_*d*_ *+X)* [23].

### Pull-down assay

RIPA buffer that contained 1% (v/v) complete protease inhibitor cocktail was added to M17-APPsw cells. The samples were centrifuged, and the supernatant was collected. Total protein levels were determined using a BCA Protein Assay Reagent kit (Rockford, IL, USA). Streptavidin beads were incubated with biotin-labeled A1, Gp30 and U31 (250 pmol each) for 30 minutes at 37°C. Washed bead-aptamer mixtures were subsequently incubated with 600 μg of cell extracts for one hour at 37°C. The mixtures were washed and heated for 10 minutes at 100°C in 50 μl of 2 x sodium dodecyl sulfate-polyacrylamide gel electrophoresis (SDS-PAGE) sample buffer. The samples were subsequently analyzed via immunoblotting. The primary antibody comprised of anti-BACE1 (Sigma Aldrich, St. Louis, MO, USA) [23].

### Fluorescence resonance energy transfer (FRET) assay

This assay was performed according to BACE1 Activity Detection Kit protocols (Sigma, St. Louis, MO). Briefly, BACE1 enzyme and substrate were diluted in a Fluorescent Assay Buffer to produce 10 x working solutions [24]. The aptamer was also diluted into different concentrations in deionized water. This assay was performed in 96-well microplates using 100 μl, which comprised of 20 μl of substrate working solution, 2 μl of BACE1 working solution, and 5 μl of different aptamer concentrations. The reaction was allowed to proceed for four hours in the dark under a lid at 37°C, and was terminated by a stop solution. The product fluorescence before and after the reaction was measured by a VICTORTM X3 multi-label plate reader (PerkinElmer, Waltham, MA, USA) using 320 nm excitation and 405 nm emission wavelengths. The percentage of inhibition was calculated using the following equation: 100-(IFi/IFo×100), where IFi and IFo represent the BACE1 fluorescence intensities in the presence and absence of inhibitor, respectively. Inhibition curves were created by graphing the percentage of inhibition versus the logarithm of the inhibitor concentration using linear regression.

### Cell viability assay

M17 cells were seeded in 96-well plates (density = 1×10^4^ cells per well). Following 24 hours of culture, cells were treated with vehicle (DMSO) or different aptamer concentrations for 24 hours in Opti-MEM. Then, the medium that contained the vehicle or aptamers was replaced with fresh medium supplemented with MTT 0.5 mg/mL in the absence of aptamers or vehicle. Cells were subsequently cultured for an additional four hours. The medium was subsequently discarded, and 100 μl of DMSO were added to the wells. Following 15 minutes of incubation with DMSO, the absorption intensities were measured at 570 nm [24].

### Quantitation of Aβ40 and Aβ42

M17 cells were seeded in 6-well plates. When the cells attained 80% confluence, cultures were refreshed for 24 hours with Opti-MEM and different A1, Gp30 and U31 concentrations. The medium was obtained from the plates to determine the secreted Aβ_40_ and Aβ_42_ levels, and a complete protease inhibitor cocktail (Rockford, IL, USA) was added with a final concentration of 1% (v/v). The medium was subsequently centrifuged at 12,000 rpm for 20 minutes at 4°C, and the supernatant was analyzed for Aβ_40_ and Aβ_42_ quantitation. To quantify the intracellular Aβ_40_ and Aβ_42_ levels, the cells were lysed as previously described. The collected cell lysates were assayed without dilution using human Aβ_40_ and Aβ_42_ double-antibody sandwich ELISA kits (Invitrogen, Carlsbad, CA, USA) [24]

### Western blotting

After 24 hours of treatment with A1 or vehicle, the M17 cells were harvested in 2 x SDS-PAGE sample buffer. The samples were resolved on 10% SDS-PAGE gels and transferred to PVDF membranes. Then, the membranes were blocked in 5% milk for one hour, followed by incubation overnight at 4°C with the corresponding primary antibody. The primary antibody was anti-APP, which is specific to the APP amino terminal (Abcam, Cambridge, UK), and anti-BACE1 (Sigma Aldrich, St. Louis, MO, USA). On the subsequent day, the membranes were washed three times with TBST buffer for five minutes. The membranes were then incubated in 5% milk (v/v) supplemented with anti-rabbit IgG or anti-mouse IgG for one hour at room temperature. Following incubation with the secondary antibody, the membranes were washed three times with TBST buffer for five minutes. The blots were then visualized via incubation with a SuperSignal West Dura chemiluminescence kit (Pierce Biotechnology, Rockford, IL, USA). Images were obtained via an AlphaImager HP System (Alpha Innotech, San Leandro, CA, USA). β-actin was used as a reference control for protein loading. The bands were quantified using Fluorchem Q SA software (Alpha Innotech, Santa Clara, CA, USA).

### Statistical analysis

Statistical analysis included one-way analysis of variance (ANOVA) for more than two groups or independent-sample *t*-tests for two groups (SPSS 13.0; SPSS Inc., Chicago, IL, USA). *K*_d_ values for each aptamer were calculated using nonlinear regression (SigmaPlot 12.0; Systat Software, San Jose, CA, USA). *P **<**0.05 and **P **<**0.01 compared with the control group were considered statistically significant.

## Results

### Aptamer selection

**Fig 1:**
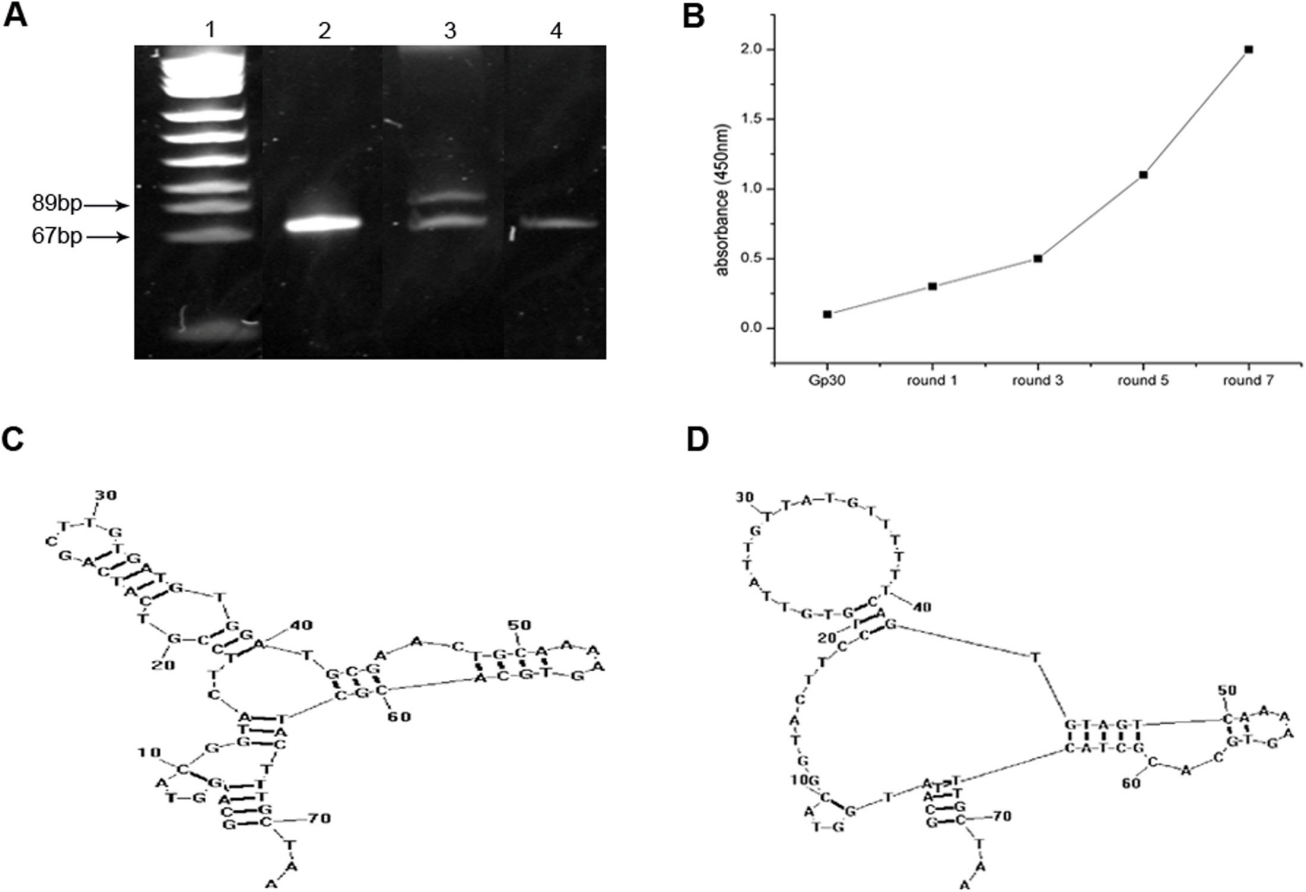
Aptamer generation using the SELEX process. (A) Seven M urea 8% denatured polyacrylamide gel electrophoresis was used to confirm ssDNA, which was separated by asymmetric PCR after each round. Lane 1: pUC18 DNA/Msp l marker. Lane 2: initial library Gp30. Lane 3: unequal length PCR products. Lane 4: target ssDNA. (B) Selected pool enrichment was monitored via indirect ELISA. (C-D) Secondary structures of aptamers A1 (C) and A4 (D) predicted by RNAstructure 5.7 software (Mathews lab, http://rna.urmc.rochester.edu/RNAstructure.html).

We used a microwell plate method to perform the SELEX procedure. For the subsequent round of selection, asymmetric PCR was conducted to separate the target ssDNA, which was identified using 7M urea 8% denatured polyacrylamide gel electrophoresis (Fig 1A). Selected pool enrichment resulted in the evolution of potential aptamer candidates, which were investigated using indirect ELISA. First, ssDNAs from each round were labeled with biotin; then, using streptavidin-conjugated horseradish peroxidase, the biotinylated aptamers reacted with BACE1 via ELISA. Initial library Gp30 and ssDNA obtained from the 1^st^, 3^rd^, 5^th^ and 7^th^ rounds were applied to perform the assay. Absorbance was increased as the selection process proceeded, which suggested enrichment in the 7^th^ round (Fig 1B). The ssDNA obtained from the final round was amplified, cloned, and sequenced. Two sequences were identified based on their primary sequence similarity and were regarded as potential aptamer candidates (A1 and A4) (Table 2). Both sequences had a primarily stem-loop structure as indicated by the secondary structure analysis (Fig 1C and 1D).

**Table 2:** Sequences of selected aptamers and *K*_*d*_ values of A1, A4 and anti-BACE1.

Name	Sequence	*Kd* (nM) ^a^
**A1**	GCAATGGTACGGTACTTCC**GTCATCAGCTTGTGATGTGGATGCGAACTG**CAAAAGTGCACGCTACTTTGCTAA ^b^	68.5655 ± 8.1237
**A4**	GCAATGGTACGGTACTTCC**TGTGTTATTGTTATGTTTTTTCAGTGTAGT**CAAAAGTGCACGCTACTTTGCTAA ^b^	15.3497 ± 2.0262
**Anti-BACE1**		2.7498 ± 0.2785

BACE1: β-Site amyloid precursor protein cleaving enzyme 1

^a^ Michaelis-Menten binding curves used to evaluate *K*_d_ (nM) were performed as described in the Materials and Methods section. Standard deviation values were determined from three independent experiments.

^b^ Bold underlined letters represent random sequences of 30 nt in length.

### Aptamers that specifically bind to BACE1 with high affinity

**Fig 2:**
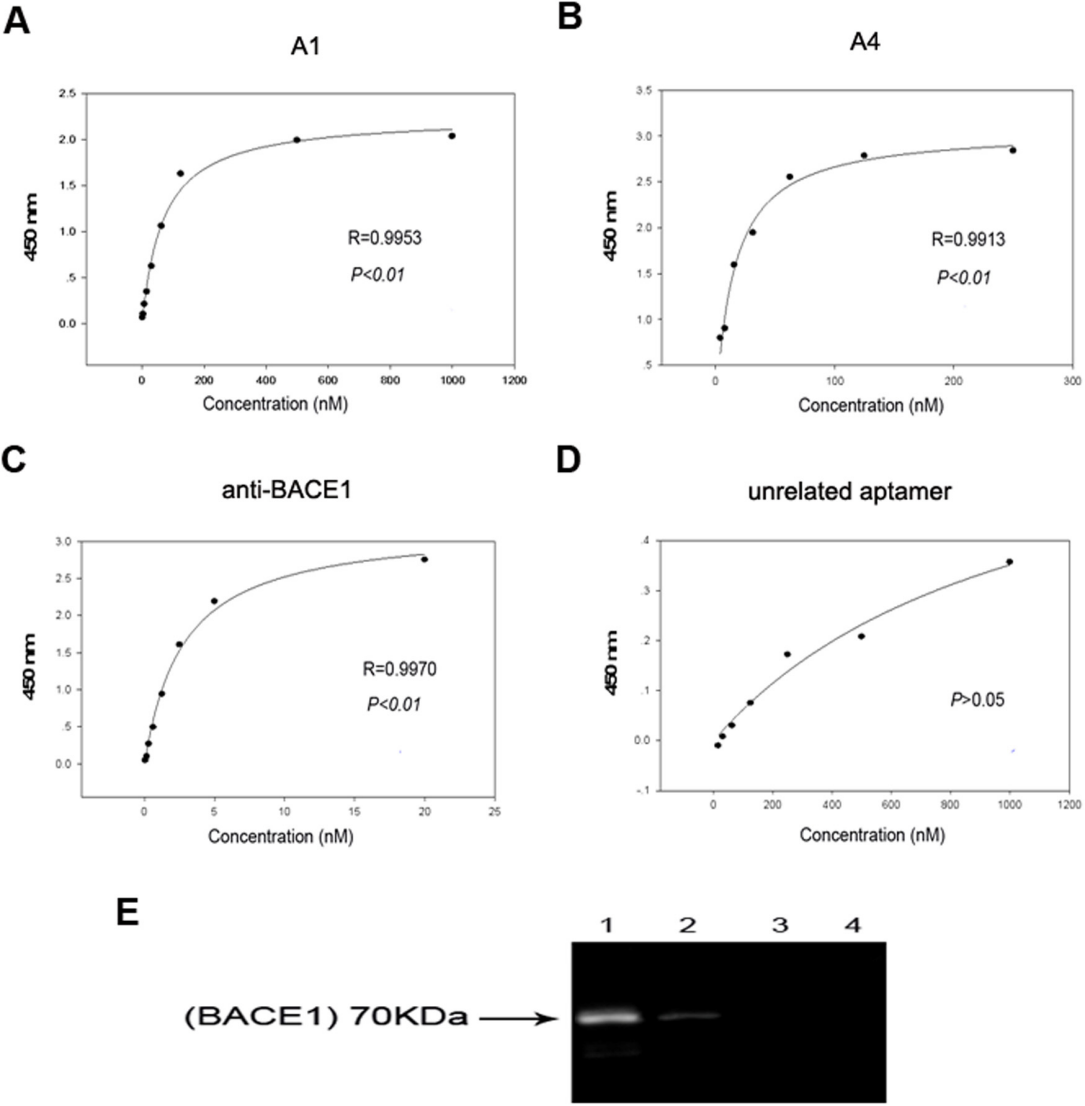
Selected aptamers to BACE1 exhibit good specificity and high affinity. (A-D) Binding curves of A1, A4, anti-BACE1 and an unrelated aptamer, U31. Mean absorbances for each aptamer concentration from three independent experiments were used to create the binding curve. (E) The specific interaction of aptamer A1 with BACE1 protein was identified via affinity purification on streptavidin beads of M17-APPsw cell lysate treated with biotin-labeled A1, followed by immunoblotting with anti-BACE1 antibody. A band at 70 kDa corresponded to the molecular weight of BACE1 and indicated that BACE1 was the specific target of the aptamer. Lane 1: A1 with M17-APPsw cell lysate. Lane 2: GP30 with M17-APPsw cell lysate. Lane 3: U31 with M17-APPsw cell lysate. Lane 4: blank control (no aptamer) group. Three independent experiments were performed.

Indirect ELISA was performed to determine the binding affinity of the selected aptamers to BACE1. Anti-BACE1 antibody and an unrelated aptamer, U31, were used as the control. Similar to anti-BACE1, the binding curves of A1 and A4 to BACE1 fit well (Fig 2A–2D) with *K*_*d*_ values in the nanomolar range (Table 2). These findings indicate that A1 and A4 can specifically bind to BACE1 with high affinity. A1 had the highest repetition frequency and was subsequently selected for the pull-down assay, which illustrated that A1 specifically interacted with BACE1 protein in M17-APPsw cells, whereas Gp30 and U31 did not exhibit binding to BACE1 proteins (Fig 2E).

### Aptamer A1 specifically inhibits BACE1 activity in vitro

**Fig 3:**
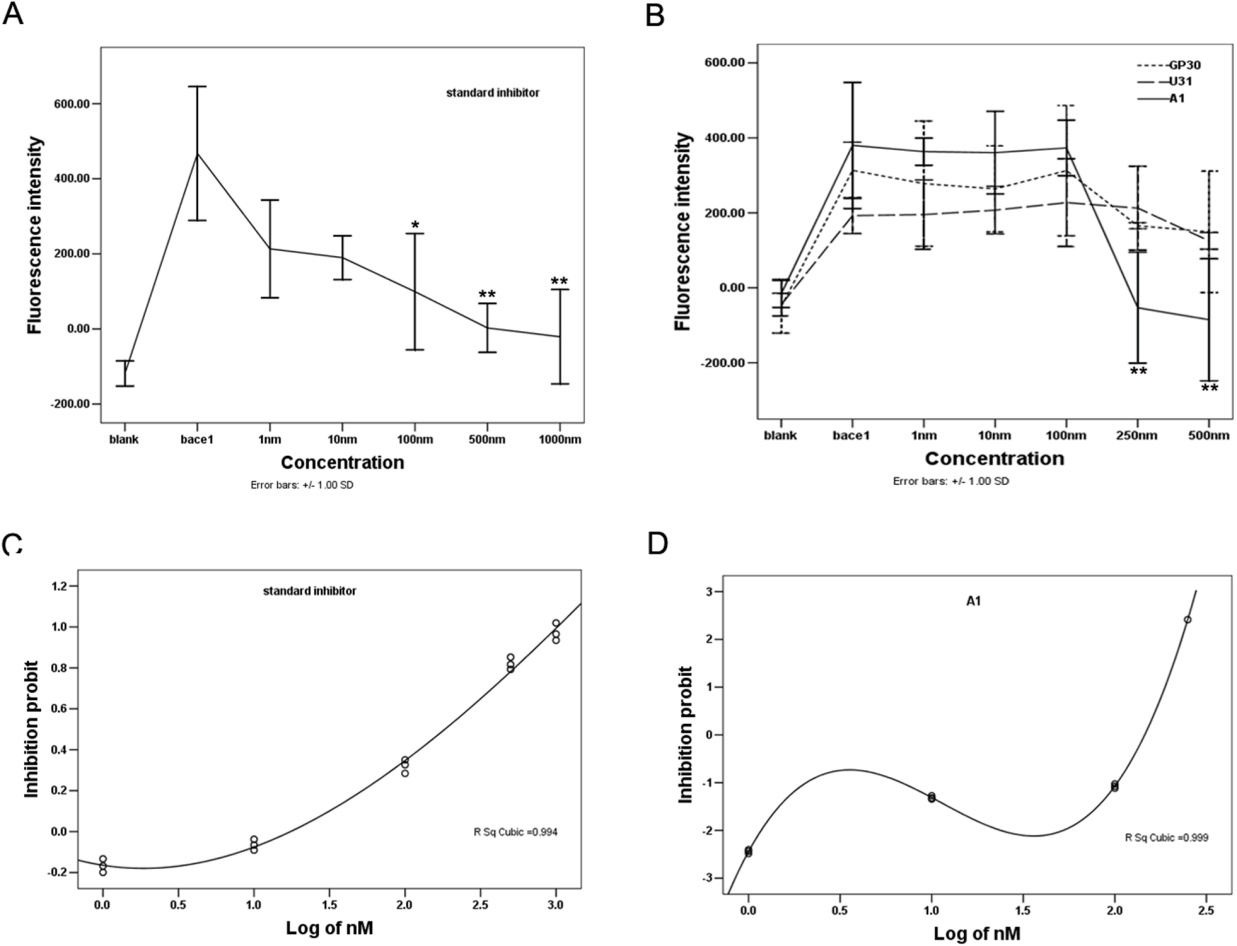
Specific inhibitory effect of aptamer A1 on BACE1 activity *in vitro*. **(**A-B**)** Effects of different concentrations of a standard inhibitor (**A**), A1 (straight line in B), Gp30 (dotted line in B), and U31 (dashed line in B) on fluorescence intensity determined by FRET assay are shown. (C-D) Inhibition profile of BACE1 activity by a standard inhibitor (C) and aptamer A1 (D) is shown. The data are represented as the mean±SD of three independent experiments. *P **<**0.05 and **P **<**0.01 compared with the positive control group.

Next, a fluorescence resonance energy transfer assay was conducted to confirm the inhibitory effect of A1 on BACE1 activity *in vitro*. The BACE1 substrate is linked to a fluorescent donor on one end and a quenching acceptor group on the other end. As a result of intramolecular energy transfer to the quenching acceptor, fluorescence from the donor can be quenched by the acceptor. Following substrate cleavage via BACE1 enzymes, the energy transfer is disturbed, which results in fluorescent signal enhancement. The fluorescence of the substrate is significantly reduced when the substrate cleavage is blocked by an inhibitor. Based on this principle, various concentrations of A1 were added into a reaction system that contained BACE1 and its fluorescent substrate; the changes in the fluorescence intensity were subsequently detected. A BACE1 specific inhibitor ([Asn670, Sta671, Val672]-Amyloid β/A4 Precursor Protein 770 Fragment 662–675), Gp30, and U31 served as controls. Following increases in the concentration, the fluorescence intensity in the standard inhibitor group gradually decreased, with a significant decrease at a concentration of 500 nM compared with the positive control (P<0.01). The fluorescence intensity in the A1 group significantly decreased at a concentration of 250 nM (P<0.01). No significant decrease in the fluorescence intensities occurred in the Gp30 or U31 groups (Fig 3A and 3B). The data of BACE1 activity were converted from percent inhibition to probit values, which were drawed versus the log of the BACE1 specific inhibitor or aptamer A1 concentration. A1 inhibited BACE1 activity with an IC_50_ value of 139.81 nM. Under the same experimental conditions, standard inhibitor inhibited the activity of BACE1 in a concentration-dependent manner (IC_50_ = 242.50 nM; Fig 3C and 3D). These findings demonstrate that A1 can act as a potent inhibitor of BACE1 activity *in vitro*.

### Aptamer A1 specifically inhibits BACE1 activity in an AD cell model

**Fig 4:**
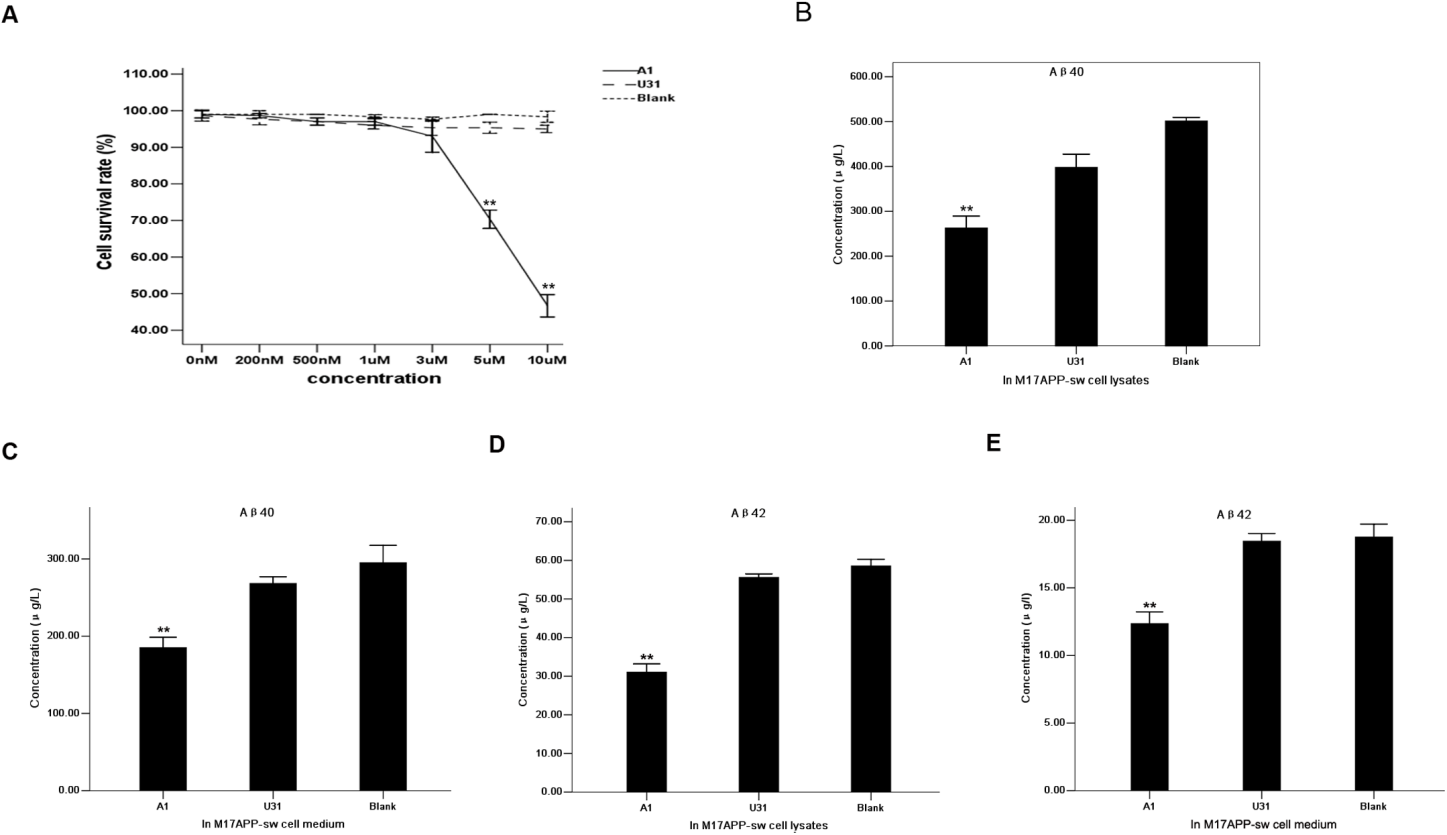
Specific inhibitory effect of aptamer A1 on BACE1 activity in an AD cell model. (A) Effects of different concentrations of A1 on M17-APPsw cell survival rate using MTT assay. A1 group: straight line. U31 group: dashed line. Blank control group: dotted line. The data are represented as the mean±SD (*n* = 3). **P **<**0.01 compared with the 0 nM group. (B-C) Comparison of Aβ_40_ concentrations secreted within M17-APPsw cells (B) and media (C) among the A1, U31 and blank control groups. (D-E) Comparison of Aβ_42_ concentrations secreted within M17-APPsw cells (D) and media (E) among these groups. In B-E, the data are represented as the mean±SD (*n* = 3). **P **<**0.01 compared with the U31 and blank control groups.

**Fig 5:**
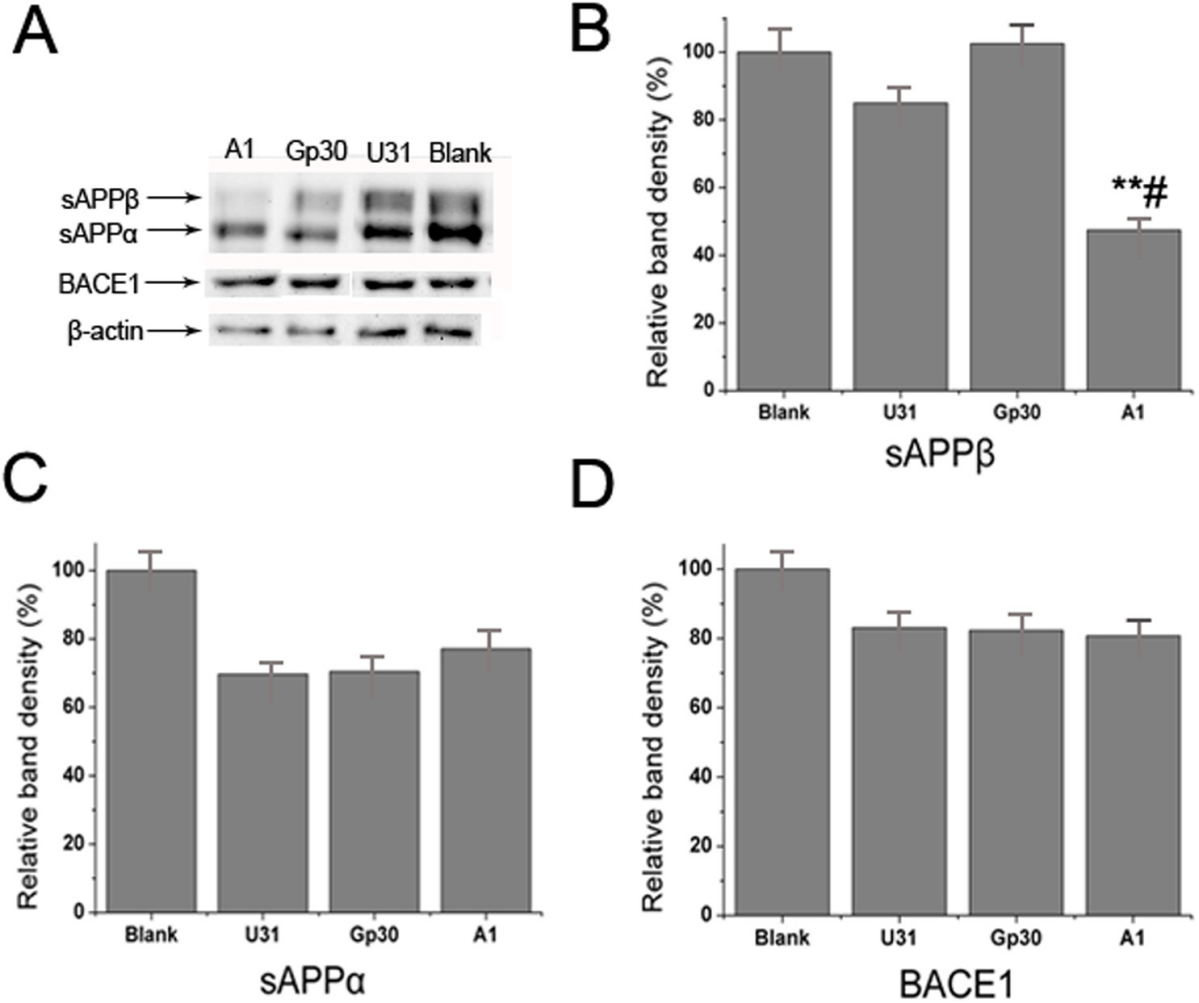
Specific inhibitory effect of aptamer A1 on BACE1 activity in an AD cell model. (A) Western blot analysis of sAPPα, sAPPβ and BACE1 expressions in the A1, Gp30, U31 and blank control groups. (B-D) Quantitative analysis of the sAPPβ (B), sAPPα (C) and BACE1 (D) bands among groups. The data are represented as the mean±SD (*n* = 3). **P<0.01 compared with the Gp30 and blank groups. ^#^ *P*<0.05 compared with the U31 group.

MTT was used to evaluate the effect of different A1 concentrations on cell survival. M17-APPsw cell viability was not affected at a final concentration of 3 μM (Fig 4A). Thus, a 3 μM A1 concentration was applied for cell incubation. The resulting cell lysates and media were resolved on a double-antibody sandwich ELISA. A 3 μM U31 treated group and a blank control (without treatment) group served as the controls. Aβ_40_ and Aβ_42_ concentrations secreted by M17-APPsw cells decreased intracellularly and in culture media compared with the control group (Fig 4B–4E). Western blot analysis indicated that sAPPβ expression was significantly decreased in the A1 treated group compared with the control group, whereas no substantial differences in sAPPα and BACE1 expression levels were identified among the groups (Fig 5). These results suggest that A1 can inhibit BACE1 activity in an AD cell model.

## Discussion

This study employed the SELEX process on a microwell plate, in which a purified extracellular region of human BACE1 protein was used as the target, and two DNA aptamers against BACE1 were identified. Since the 4^th^ round, a blank well was introduced for counter selection to exclude the enrichment of nonspecific sequences. Although many modified SELEX methods have been developed such as capillary electrophoresis SELEX and whole-cell SELEX, traditional SELEX technology possesses the superiority of rapid and simple operation, as well as excellent specificity of selected aptamers to target proteins. The fitted curves and low *K*_d_ values of these aptamers, which were determined by indirect ELISA, indicate high affinity and specificity to BACE1.

Aptamers to proteins often bind in functionally important parts of a molecule, such as a substrate binding pocket or allosteric site, to modulate physiological function [25]. Aptamers can execute their functions by directly binding to the target protein without affecting its gene expression, in contrast to other small noncoding RNAs that inhibit gene expression such as microRNAs, siRNAs and antisense [26]. Aptamers exhibit direct effects for treating diseases both within and outside the CNS [27, 28]. The FRET assay demonstrated the strong inhibitory effect of A1 on BACE1 activity *in vitro*, in which the IC_50_ value of A1 was smaller than a standard inhibitor. We speculate that there may be sequence specificity between A1 and the catalytic site of BACE1, which thus enables A1 to bind and interfere with BACE1. It may be possible to truncate part of the aptamer’s sequence to determine the functional regions of A1. Although RNA aptamers specific to the cytoplasmic domain of BACE1 have been reported [29, 30], these aptamers only provide a useful tool to elucidate the functions of the short cytoplasmic tail of BACE1. These aptamers are not suitable for AD drug development, because the enzymatic activity of BACE1 resides in the extracellular domain and the cytoplasmic domain of BACE1 is difficult to bind to aptamers without the facilitation of other carriers.

In addition, the uncompromising nature of the protease active site makes it difficult to manipulate inhibitor structures critical for improved drug properties [30]. Major obstacles in BACE1 inhibitor development were low oral bioavailability and restricted brain exposure caused by affinity for drug transporter proteins, which result in low drug concentrations at the target site [31, 32]. Intrinsic characteristics of aptamers make them easy to incorporate into biocarriers to penetrate the BBB and improve target delivery [33, 34]. Furthermore, aptamers have unique properties; suggesting that they are promising tools for the diagnosis and treatment of various diseases. Aptamers can discriminate between different conformations of the same target protein, and exhibit their capability to quantify the expression level of different protein forms in diagnostic applications. They can also exhibit a potential interference with the target product in therapeutic applications [26]. Decreased Aβ_40_ and Aβ_42_ production and sAPPβ levels following A1 treatment in M17-APPsw cell cultures illustrate the impressive inhibitory effects of A1 on BACE1 activity, which supports the preliminary possibility that A1 could represent a potential BACE1 inhibitor. This finding requires further confirmation via *in vivo* experiments in an AD animal model, and the potential side effects of A1 must also be determined. Western blot analysis indicated the sAPPα levels were not affected by A1. This may be due to the impact on the α-pathway after partial β-pathway inhibition, which was too subtle to alter sAPPα levels; and majority of APPs were cleaved by α-secretase.

Several factors should be considered in applying aptamers *in vivo* such as the degradation of unmodified nucleic acids, short shelf-life, and ability to penetrate the BBB. There are several ways to improve chemical stability and extend the half lives of aptamers by modification *in vivo* (such as base modification, backbone modification, and PEG modification). Some aptamers that could penetrate the BBB were screened via *in vivo* SELEX. Aptamers can also be delivered across the BBB by liposomes, nanoparticles and protein transduction domains [34]. In future experiments, we plan to determine the effects of these aptamers in AD models and explore the method and mechanism of aptamer delivery across the BBB, as well as their effects in AD animal models. We will express a series of proteins according the catalytic site of the BACE1 protein, including point mutant or domain-negative peptides or proteins, EMSA or a pull down assay will subsequently be applied to determine the aptamer A1 interactions with expressed and purified peptides and to identify the important amino acids or peptides in the catalytic site and molecular simulation using software.

In conclusion, this preliminary study regarding aptamer identification using a SELEX strategy provides novel evidence that aptamers have the ability to inhibit BACE1 activity in an AD cell model and supports its potential in the development of a novel and specific BACE1 inhibitor.
